# A Case of Pseudohyponatremia in the Setting of Pegaspargase-Induced Hypertriglyceridemia in an Adult T-Cell Lymphoblastic Leukemia Patient

**DOI:** 10.1155/crh/7154679

**Published:** 2024-12-29

**Authors:** Asim Ahmad, Matthew Joseph

**Affiliations:** ^1^Cancer, and Texas College of Osteopathic Medicine, The Center for Cancer and Blood Disorders, Fort Worth, Texas, USA; ^2^Texas College of Osteopathic Medicine, University of North Texas Health Science Center, Fort Worth, Texas, USA

**Keywords:** adult T-cell acute lymphoblastic leukemia, hypertriglyceridemia, pegaspargase, pseudohyponatremia

## Abstract

We present a rare case of pseudohyponatremia in a 20-year-old male patient with adult T-cell acute lymphoblastic leukemia (ATLL). The patient was admitted for a mediastinal mass with superior vena cava syndrome and was receiving pegaspargase therapy. The pseudohyponatremia was found to be secondary to hypertriglyceridemia associated with the pegaspargase treatment. In this case report, we discuss the important considerations in the management of adult ATLL patients receiving pegaspargase therapy, and the specific approaches taken to care for the patient described in this case.

## 1. Introduction

ATLL is a rare and aggressive malignancy associated with human T-cell lymphotropic virus type 1 (HTLV-1) infection. Pegaspargase, an enzyme used in the treatment of acute lymphoblastic leukemia (ALL), is also utilized in treating ATLL due to its ability to deplete asparagine, an amino acid crucial for the survival of malignant lymphoid cells. However, pegaspargase therapy is known to have several side effects, including hepatotoxicity, coagulopathy, pancreatitis, hyperglycemia, thrombosis, transaminitis, hyperbilirubinemia, and hypertriglyceridemia [1, 2].

Hypertriglyceridemia is a known but rare complication of pegaspargase therapy, and it is important to account for this due to the risk of acute pancreatitis, another major complication of the treatment. In the product information for pegaspargase, common side effects include hypersensitivity reactions, coagulopathy, liver dysfunction, and pancreatitis. Although hypertriglyceridemia is infrequently reported, it can lead to pseudohyponatremia, an artifactually low serum sodium concentration (SSC) due to the displacement of aqueous sodium by lipids in the plasma [3–5].

This case report aims to highlight the occurrence of pseudohyponatremia secondary to hypertriglyceridemia in a patient undergoing pegaspargase therapy for ATLL and discuss the management strategies employed.

## 2. Case Presentation

A 20-year-old male presented with symptoms of cough, dyspnea, and swelling of the face and neck. Physical examination revealed a mediastinal mass, and imaging studies, including a computed tomography angiography (CTA), confirmed the presence of a large mass causing superior vena cava syndrome. The patient was diagnosed with ATLL based on biopsy results. These complications were secondary to delay in therapy, resulting in this extreme presentation. General presentation involves only mild adenopathy and cytopenia within this population.

The patient was initiated on the hyper-CVAD chemotherapy regimen, which included pegaspargase. Treatment protocol aligns with GRAALL-LYSA LL03 study design which dosed patients aged 18 to 50 with newly diagnosed and previously untreated lymphoblastic leukemia. Treatment consisted of a corticosteroid prephase along with a five drug induction and giving sequential cyclophosphamide, dose-dense consolidation, late intensification, central nervous system prophylaxis with a follow-up two-year maintenance phase [6]. During the course of treatment, laboratory tests revealed severe hypertriglyceridemia (triglyceride level: 2500 mg/dL) and hyponatremia (serum sodium: 125 mEq/L). On physical examination, we diffuse adenopathy, hepatosplenomegaly, fever, and chills, and significant changes to baseline weight were present. Complete blood count drawn indicated leukocytosis, thrombocytopenia, and anemia, expected for the given diagnosis. Given the clinical context and laboratory findings, pseudohyponatremia was suspected.

Further diagnostic workup, including serum osmolality measurement and lipid panel, confirmed the diagnosis of pseudohyponatremia secondary to hypertriglyceridemia induced by pegaspargase. Prior echocardiogram was taken for heart status before starting doxorubicin, and abdominal ultrasound assessed liver architecture before central port placement through hepatic vein. This port was related to mediastinal mass resulting in congestion that did not allow for subclavian vein placement. The patient was managed with lipid-lowering agents and dietary modifications, which successfully reduced triglyceride levels and normalized serum sodium levels.

Specific interventions included manually correcting sodium due to the elevated triglycerides and to remain in normal range on resolution of the cancer (Table 1).

## 3. Case Summary

The patient was a 20 year old initially admitted for a mediastinal mass, treated for symptoms of superior vena cava syndrome and tracheal stenosis, with ATLL associated pancytopenia. ATLL is a difficult disease to treat, but the utilization of pediatric regimens for ATLL has been an important step in improving progression-free survival (PFS) and overall survival (OS).

Utilizing pediatric regimens for treating T-cell ALL (T-ALL) has significantly improved PFS and OS for adult patients, contrasting with historically high mortality rates associated with adult regimens like hyper-CVAD, which are linked to substantial toxicity and adverse effects, particularly in older adults. Modifications to hyper-CVAD aim to reduce toxicity while maintaining efficacy, but retrospective studies indicate varying CR rates (81.7%–84.28%) and 5-year OS (38%–51.7%), with chemotherapy-related complications contributing significantly to mortality. In contrast, the DFCI ALL Consortium Protocol 00-01, involving induction with vincristine, prednisone, doxorubicin, methotrexate, cytarabine, and L-asparaginase, shows promising 5-year EFS rates of around 90%. A retrospective analysis comparing DFCI and hyper-CVAD in patients under 50 years old reveals better 3-year LFS and OS with the pediatric-inspired regimen (up to 72.6% vs. 48.5% with hyper-CVAD), albeit with associated toxicities such as pancreatitis and osteonecrosis. While pediatric protocols demonstrate feasibility and efficacy in younger adults with ALL, further investigation through randomized controlled trials is warranted to optimize treatment strategies and outcomes [5–8].

Comparing the hyper-CVAD regimen and DFCI regimen for treating ALL in adolescent and young adult (AYA) patients reveals significant differences in terms of toxicity profiles and treatment-related outcomes. While both therapies offer increased benefits, they also come with respective toxicities. Hyper-CVAD regimens are associated with higher rates of myelosuppression, infection, hepatic dysfunction, infertility, cardiotoxicity, and secondary malignancies, leading to prolonged hospitalization and increased treatment-related mortality rates. In contrast, the DFCI regimen shows lower treatment-related mortality and reduced hospitalization, suggesting better overall outcomes and improved patient well-being. However, various studies on the DFCI protocol emphasize the observation of hypersensitivity reactions to asparaginase therapy, pancreatitis, and thrombotic events, particularly associated with PEG-asparaginase, though limited in number should be acknowledged in the use of this regimen [8, 9].

Hypertriglyceridemia, a more infrequent side effect of pegaspargase, became evident in this case, leading to pseudohyponatremia. This complication, as observed in our patient's blood sample causing interference with electrolyte analysis, aligns with findings from limited case reports [10, 11]. The interplay between hypertriglyceridemia and electrolyte abnormalities underscores the importance of recognizing and managing these complications to avoid further serious consequences, such as acute pancreatitis.

The identified hypertriglyceridemia, although rare, should prompt clinicians to consider alternative electrolyte analysis methods, such as direct ion-specific electrode methodology, to prevent inaccuracies in results. Furthermore, close monitoring and prompt intervention are crucial in managing hypertriglyceridemia to prevent complications like pancreatitis. Treatment may involve insulin administration, dietary restriction, omega-3, and, in extreme cases, interventions like plasmapheresis or fibrates [12, 13]. Fibrates have shown improved clinical benefits versus omega-3 for patients diagnosed with hypertriglyceridemia, showing in one randomized placebo controlled trial to have a 29% reduction in triglyceride values compared to 21% reduction in omega-3s [14].

### 3.1. Hyponatremia vs Pseudohyponatremia

A challenge that presents to providers is the mixed nature of the presentation of hyponatremia when treating for acute lymphoma [15].

Hyponatremia is a condition characterized by low levels of sodium in the blood. The normal range for serum sodium levels is 135–145 mEq/L. This imbalance can be caused by various factors affecting sodium and water regulation in the body. Key regulators include aldosterone, natriuretic peptides, and antidiuretic hormone (ADH). Symptoms of hyponatremia are diverse and can include concentrated urine, headache, nausea, vomiting, confusion, and, in severe cases, seizures and coma.

Notably, hyponatremia is often associated with an associated low “measured” serum osmolality, termed hypotonic hyponatremia. Measured serum osmolality is a crucial diagnostic marker and is calculated as 2 times sodium concentration in mEq/L plus contributions from glucose and blood urea nitrogen. Rapid correction of hyponatremia can lead to osmotic demyelination syndrome (ODS), which can have severe neurological consequences. It is important to distinguish between acute and chronic hyponatremia for appropriate management [12–14].

Pseudohyponatremia is a phenomenon where the measured sodium concentration ([Na]S) in mEq/L appears lower than its actual value due to various mechanisms. The relationship between SSC in mEq/L and serum water concentration (SWC) is expressed as SSC + SWC = 1. The SWC, slightly lower than 1—SSC, includes molecular volumes of crystalloids in addition to water volume. Pseudohyponatremia occurs when [Na]S is measured indirectly (e.g., via indirect ion-selective electrode (ISE) or flame emission spectrometry (FES)) and involves mechanisms such as the electrolyte exclusion effect, dilution effect, and hyperviscosity effect. These mechanisms are absent when using direct ISE. The electrolyte exclusion effect, leading to lower [Na]S, is explained by a decrease in electrolyte concentrations in serum due to their containment only in the SWC. The phenomenon is influenced by SSC in mEq/L and SWC, with underestimation increasing as SSC rises and SWC decreases. Disease states associated with pseudohyponatremia include diabetic ketoacidosis, hyperproteinemia, and hypertriglyceridemia as was in this case [12, 15–17].

Managing pseudohyponatremia involves addressing the challenges associated with inaccurate sodium concentration measurements, particularly when using the indirect ISE method.

Current practice for identifying whether the value is pseudohyponatremia includes the use of indirect ion specific electrodes (ISEs) for the estimation of serum sodium in mEq/L due to its use in many autoanalyzers common in care settings. A study by Aziz et al. suggests that clinicians should be aware of their laboratory's method for measuring SSC ([Na]S) in mEq/L. If a direct ISE is utilized, the [Na]S result can be accepted at face value. However, when an indirect ISE or FES is employed, further examination is necessary if [Na]S is lower than 137 mmol/L [15, 18, 19].

To correctly manage pseudohyponatremia, the passage recommends measuring serum proteins and lipids along with [Na]S. This additional information assists in calculating SSC and SWC ([Na]SW). The identification of pseudohyponatremia should shift the focus of management from hyponatremia to the underlying condition causing the elevated SSC. It emphasizes the potential risks of mismanagement, including severe neurological manifestations and deaths, resulting from inappropriate treatments such as fluid restriction and saline infusion [16, 17, 20, 21].

### 3.2. Study Limitations

Some limitations with this study included the limited sample size of one. For proper treatment of this disease, randomized controlled trials with varied chemotherapy dosing and patient characteristics are required to determine how aggressive the treatment needs to be while accounting for potential complications of therapy.

## 4. Conclusion

Pseudohyponatremia is a known complication in patients with elevated triglycerides unrelated to sodium stores of patients and can be commonly confused with Syndrome of Inappropriate ADH Secretion (SIADH) in the setting of a hyponatremia with an ongoing disease process while in the hospital [22]. Elevated triglycerides, while a rarer complication of pegaspargase compared to L-asparaginase, can result in elevated risk of acute pancreatitis and can postpone therapy. Hyponatremia is commonly an indication to keep a patient within the hospital and can both increase time before therapy and increase potential costs to both the patient and hospital system if not accounted for in the care process.

The patient's morbidity due to the mediastinal mass was extensive on initial workup, and delay of therapy he was to receive outpatient only increased the risk of continued development of this morbidity. In addition, his concurrent complications related to his disease state provided increased difficulty in determining whether his ongoing hyponatremia was a result of various factors related to his care, or due to the treatment provided.

This case emphasizes the need for healthcare providers to be vigilant in monitoring and managing complications associated with pegaspargase therapy, especially in adult patients with ATLL. It underscores a need for clinicians to be aware of uncommon side effects, consider alternative electrolyte analysis methods, and actively manage hypertriglyceridemia to prevent severe consequences, such as acute pancreatitis. Furthermore, the case highlights the mixed nature of hyponatremia in the context of acute lymphoma treatment, involving dilutional hyponatremia and hyperglycemia, necessitating careful diagnostic workup to distinguish between various causes, such as SIADH and pseudohyponatremia.

When looking at the case in this study versus others in previous literature, one review of various well known case studies for ATLL treatment looked at various factors to consider for best treatment practices for ATLL. The age ranges for these cases were older, ranging between 48 and 67 and of various ethnic backgrounds commonly endemic HTLV-1. When reviewing comparisons of treatment methodologies, two cases looked at older patients who received more intensive therapies, including cyclophosphamide, hydroxydaunorubicin, oncovin/vincristine sulfate and prednisone (CHOP) and cyclophosphamide, vincristine sulfate, doxorubicin/adriamycin and dexamethasone (hyper-CVAD), the latter of which is closest to that completed in this study. It was found that in the patients this was completed with, higher risks of complications were present. Particularly, myelosuppression, infection, and organ toxicity were typically found. More conservative therapies, such as the use of zidovudine with interferon alpha (ZDV/IFN-alpha) in older populations caused fatigue and systemic symptoms leading to compliance issues among two older patients treated. OS, while not measured in the case of this study, was found to hold remission in both aggressive therapy cases and limitations to PFS with both patients in the latter two cases treated with conservative therapy. Eastern Cooperative Oncology Group (ECOG) performance status also guided what therapy was provided in each of these four cases. Those with better overall wellness and ability to do activities of daily living were determined eligible for more intensive therapy regimens, and those with already poor ability to complete activities of daily living were given more conservative treatments. This was not determined with the patient in this study, but the side effect profile of the patient matched what was found in the case reports in review [23].

Understanding the mechanisms of pseudohyponatremia, the potential pitfalls of aggressive therapy, and looking at each patient individually when considering potential risks is crucial for accurate diagnosis and management. The interplay of electrolyte exclusion effect, dilution effect, and hyperviscosity effect can lead to falsely low sodium values in indirect measurements. Clinicians are advised to be familiar with their laboratory's measurement methods, with a preference for direct ion-specific electrode methodology.

## Figures and Tables

**Table 1:** Comprehensive metabolic panel after completion of AALL0434 regimen.

	Value	Reference ranges and units
Sodium	126(L)	136–145 mmol/L
LDL calc	367(H)	0–100 mg/dL
Triglyceride	1352(H)	0–150 mg/dL
Cholesterol	648(H)	>−200 mg/dL
Serum osmolality	264	
Lipase	42	

*Note:* Source: patient chart.

## Data Availability

Patient Health Information supporting this case report is available upon request from the corresponding author, and all other relevant data are included within the manuscript. Data relevant to the case are available upon request to the medical records department of The Center for Cancer and Blood Disorders Registered OSF registries under osf.io/6tdn2.
